# Older Adults’ Daily Step Counts and Time in Sedentary Behavior and Different Intensities of Physical Activity

**DOI:** 10.2188/jea.JE20200080

**Published:** 2021-05-05

**Authors:** Shiho Amagasa, Noritoshi Fukushima, Hiroyuki Kikuchi, Koichiro Oka, Sebastien Chastin, Catrine Tudor-Locke, Neville Owen, Shigeru Inoue

**Affiliations:** 1 Department of Preventive Medicine and Public Health, Tokyo Medical University, Tokyo, Japan; 2 Faculty of Sport Sciences, Waseda University, Saitama, Japan; 3 School of Health and Life Science, Institute of Applied Health Research, Glasgow Caledonian University, Glasgow, United Kingdom; 4 Department of Sport and Movement Science, Ghent University, Ghent, Belgium; 5 Department of Kinesiology, School of Public Health and Health Sciences, University of Massachusetts Amherst, MA, USA; 6 Behavioral Epidemiology Laboratory, Baker Heart and Diabetes Institute, Melbourne, Australia; 7 Centre for Urban Transitions, Swinburne University of Technology, Melbourne, Australia

**Keywords:** accelerometry, exercise, walking, ambulatory, sedentary lifestyle

## Abstract

**Background:**

Daily step count is the simplest measure of physical activity. However, little is known about how daily step count related to time spent in different intensities of physical activity (PA) and sedentary behavior (SB).

**Methods:**

These cross-sectional data were derived from 450 older Japanese adults (56.7% men; mean age, 74.3 years) who were randomly selected from three communities and responded a survey. Daily step count and time spent in moderate-to-vigorous PA (MVPA), light-intensity PA (LPA), and SB were measured using a validated wearable technology (HJA-350IT). Associations of daily step count with time spent in measured behaviors were examined using linear regression models with isometric log-ratio transformations of time-use composition, adjusting for gender, age, and residential area.

**Results:**

Participants averaged 5,412 (standard deviation, 2,878) steps/d and accumulated MVPA, LPA, and SB corresponding to 4.0%, 34.8%, and 61.2% of daily waking time, respectively. Daily step count significantly increased with increase in time spent in MVPA relative to other behaviors (ie, LPA and SB) and in the ratio of LPA to SB after allowing for MVPA. After stratification, daily step count was significantly related to the ratio of LPA to SB in those taking <5,000 steps/d, but not in those taking 5,000–7,499 and ≥7,500 steps/d.

**Conclusions:**

Higher daily step count can be an indicator of not only larger relative contribution of time spent in MVPA, but also higher ratio between LPA and SB, particularly among those who are the least physically active.

## INTRODUCTION

Walking is the most popular form of aerobic physical activity.^1^ Evidence on walking and health outcomes shows higher daily step count to be associated with a lower risk of all-cause mortality^2^^–^^5^ and better cardiovascular and bone health.^6^^,^^7^ A recent meta-analysis of 37 randomized controlled trials concluded that there are consistently favorable effects of walking interventions on cardiovascular disease risk factors among inactive healthy adults.^8^ Increasing walking provides a readily-accessible means of promoting health benefits.^9^

Daily step count is a simple and direct measure of physical activity volume, and is also associated with time spent in moderate-to-vigorous physical activity (MVPA).^10^ However, little is known how daily step count is related with time in the other behaviors, including light-intensity physical activity (LPA) and sedentary behavior (SB), which can be important for health. A body of literature indicates beneficial effects of LPA and determinantal effects of SB.^11^^–^^13^ Higher daily step count may coexist with a physically active lifestyle associated with more time spent in LPA and less time spent in SB.^14^ Another possibility is that individuals are more likely to be sitting (and therefore accumulating more time in SB) to take a rest if they do a lot of walking. There is a need to see if daily step count can be used for indirect estimates of time spent in SB and different intensities of physical activity to communicate these behavior data. One previous study has shown adult’s daily step count has strong associations with LPA and moderate-intensity physical activity and a moderate association with SB.^10^ However, this study did not fully consider the compositional nature of time-use data.

Time is finite. If time spent in one behavior is changed, it will necessarily influence the time spent in other behaviors. Compositional data analysis (CoDa) allows consideration of the inherent dependency of time spent in all behaviors arising within a day or part of a day.^15^^,^^16^ Therefore, the present study examined how daily step count is related to time spent in different intensities of physical activity and SB during the day, using the CoDa approach. Our hypothesis is that daily step count is related not only to the relative proportion of time spent in MVPA, but also to time spent in more LPA and less SB, after controlling for time spent in all behaviors.

## METHODS

### Study sample and data collection

This secondary data analyses was based on cross-sectional data collected in 2015. Participants included a subset of older adults who responded to a population-based survey in 2010. Briefly, in 2010, a total of 2,700 residents living in Japanese cities (Bunkyo [urban], Fuchu [suburban], and Oyama [rural]) were recruited using stratified random sampling from a resident registry. The sampling process and recruitment strategy for the 2010 survey and characteristics of each area are described elsewhere.^17^ In 2015, 1,210 participants (70–79 years of age) who agreed to participate received a mailed questionnaire, and 988 completed and returned the survey.^18^ Of these respondents, 478 agreed to wear an accelerometer. Accelerometers with written instructions were delivered and returned via mail. The Tokyo Medical University Ethics Committee (No. 2898) granted ethical approval prior to the survey. Written informed consent was obtained from all participants.

### Accelerometer-assessed activity measures

Daily step count and time in different intensities of physical activity and SB was measured using the Active style Pro HJA-350IT (Omron Healthcare, Kyoto, Japan). Participants were asked to wear an accelerometer on their waist for 7 consecutive days while awake except for water-based activities (eg, swimming). Active style Pro is a validated accelerometer^19^^–^^21^ that provides data comparable to the devices most commonly used in studies conducted in Western countries.^22^^,^^23^ Its measurement algorithm has been described in detail elsewhere.^19^^,^^20^ We used 1-minute epoch data and obtained time spent in categories bound by estimated metabolic equivalents (METs) values. A METs-based cutoff was used to determine intensity of activities: ≤1.5 METs for SB, 1.6–2.9 METs for LPA, and ≥3.0 METs for MVPA.^24^^,^^25^ No acceleration signal detected for longer than 60 consecutive minutes was defined as “non-wear”, and only records from participants wearing the accelerometer for at least 10 hours per day were considered valid.^26^ Participants with data from at least 4 valid days^27^^,^^28^ were included in the analysis.

### Questionnaire data

Gender and age were obtained from the original resident registry. Participants reported their height, weight, living arrangement, working status, physical limitations, and self-rated health. Body mass index (BMI) was calculated from height and weight (kg/m^2^). Perceived health and physical limitation were evaluated with items from the SF-8 (Japanese version).^29^ Responding to the question; “Overall, how would you rate your health during the past 4 weeks?”, participants chose the answer that most accurately described their health on a 6-point scale: excellent, very good, good, fair, poor, and very poor.^29^ The answers were classified into “good” (excellent, very good, and good) and “poor” (fair, poor, and very poor). Responding to the question; “During the past 4 weeks, how much did physical health problems limit your physical activities (such as walking or climbing stairs)?”, participants chose the answer that most accurately described their physical limitation on a 5-point scale: not at all, very little, somewhat, quite a lot, and could not do physical activities.^29^ The answers were classified into “no” (not at all and very little) and “yes” (somewhat, quite a lot, and could not do physical activities).

### Statistical analyses

R version 3.5.3 (R Foundation for Statistical Computing, Vienna, Austria) was used to perform all statistical analyses; a *P*-value of <0.05 was regarded to be a statistically significant difference. The “Compositions” package was used to analyze data.

We characterized time spent in different intensities of physical activity and SB, based on previously suggested graduated categories for daily step counts: <5,000, 5,000–7,499, and ≥7,500 steps/d.^30^ A ternary diagram was used to describe sample distributions of time spent in different intensities of physical activity and SB. Multivariate analysis of variance was performed to test whether timed-stamped intensities differed according to daily step count categories.

To investigate how daily step count relate to time-stamped intensities, we performed CoDa, as detailed in previous research.^15^^,^^16^ First, geometric (compositional) means were calculated so that all contributing categories ultimately added up to 100%, representing accelerometer wear time. We chose not input non-wear time into the composition as a means of focusing on the waking day and avoiding introducing more noise in the models. Composition does not have to be on an invariant fixed sum.^31^ Variability in the data, in terms of variability of each behavior relative to the variability of other behaviors, was described through a variation matrix. Second, we conducted isometric log-ratio (ilr) transformation to introduce the full composition into the models and adequately adjust the models for time spent in the other behaviors. The log-ratio methodology allows us to apply compositional data to standard statistical methods. No statistical process was required to handle zero values, as all participants spent some time in every time-stamped intensity category. Below are formulas of ilr transformation where MVPA time segments are considered first. The ilr coordinate1 presents the ratio of MVPA time to time spent in all other intensities of physical activity and SB, whereas ilr coordinate2 expresses the ratio of LPA time to SB time.

$$\text{ilr coordinate1}=\sqrt{\frac{2}{3}}\text{ln}\frac{\text{MVPA}}{\sqrt{\text{LPA}\times \text{SB}}}$$

$$\text{ilr coordinate2}=\sqrt{\frac{1}{2}}\text{ln}\frac{\text{LPA}}{\text{SB}}$$

Linear regression models were used to examine 1) the relationships between daily step count and time spent in time-stamped intensity categories (R^2^adj = 0.62), and 2) how daily step count is predicted by the specific time-stamped intensity categories contributing to the composition and then 3) map area of daily step count on a ternary diagram to show what categorical component of the composition is likely to be related to daily step count, adjusting for age, gender and city of residence.

## RESULTS

### Participant characteristics

In total, 450 participants (56.7% men) with a mean age of 74.3 (standard deviation [SD], 2.9) years provided valid data and were included in these analyses. The majority reported being living with others (85.8%), and in good perceived health (80.7%) and no physical limitation (77.3%) (Table 1). Significant differences between graduated daily step count categories were observed by gender, city of residence, working status, physical limitations, and perceived health.

**Table 1.** Characteristics of study participants and time spent in intensities of physical activity and sedentary behavior by step-based categories

	Overall	<5,000 steps/d	5,000–7,499 steps/d	≥7,500 steps/d	*P*-value
	(*n* = 450)	(*n* = 224)	(*n* = 121)	(*n* = 105)	

	*n* (%)/mean (SD)	*n* (%)/mean (SD)	*n* (%)/mean (SD)	*n* (%)/mean (SD)	
Gender, men	255 (56.7%)	114 (50.9%)	77 (63.6%)	64 (61.0%)	**0.045**^a^
Age, years, mean (SD)	74.3 (2.9)	74.3 (2.9)	74.7 (2.7)	74.0 (2.9)	0.119^b^
City of residence, urban	142 (31.6%)	66 (29.5%)	32 (26.4%)	44 (41.9%)	**0.028**^a^
Working status, yes	131 (29.1%)	51 (22.9%)	47 (38.8%)	33 (31.7%)	**0.006**^a^
Living arrangement, with others	386 (85.8%)	191 (85.7%)	109 (90.1%)	86 (81.9%)	0.207^a^
Body mass index, kg/m^2^, mean (SD)	22.5 (3.0)	22.4 (3.2)	22.9 (2.8)	22 (12.6)	0.104^b^
Perceived health, good	363 (80.7%)	165 (73.7%)	102 (84.3%)	96 (91.4%)	**0.005**^a^
Physical limitation, no	348 (77.3%)	160 (72.7%)	97 (80.8%)	91 (87.5%)	**0.006**^a^
Step count, steps/d, mean (SD)	5,412 (2,878)	3,106 (1,222)	6,124 (707)	9,512 (1,714)	
Accelerometer wear time, min/d,^*^ mean (SD)	873.9 (90.4)	865.4 (91.0)	872.8 (90.7)	893.3 (87.5)	**0.031**^b^
SB, min/d, mean (SD)	521.7 (119.5)	552.7 (113.7)	496.4 (120.0)	484.9 (114.4)	**<0.001**^b^
LPA, min/d, mean (SD)	307.4 (102.0)	287.7 (102.3)	324.6 (93.5)	329.5 (103.6)	**<0.001**^b^
MVPA, min/d, mean (SD)	44.8 (31.3)	25.0 (17.9)	51.8 (24.7)	78.9 (27.6)	**<0.001**^b^
Proportion of accelerometer wear time					
SB, %	61.2	65.4	57.4	55.0	**<0.001**^c^
LPA, %	34.8	32.4	37.1	36.4	
MVPA, %	4.0	2.3	5.6	8.7	

Values reported as *n* (%) unless otherwise noted.

LPA, light-intensity physical activity; MVPA, moderate-to-vigorous physical activity; SB, sedentary behavior; SD, standard deviation.

^a^Chi-squared test.

^b^Analysis of variance.

^c^Multivariate analysis of variance.

^*^Arithmetic mean.

Missing value: working status; *n* = 2, living arrangement; *n* = 1, and physical limitation; *n* = 6.

### Descriptive statistics

**Figure 1.**
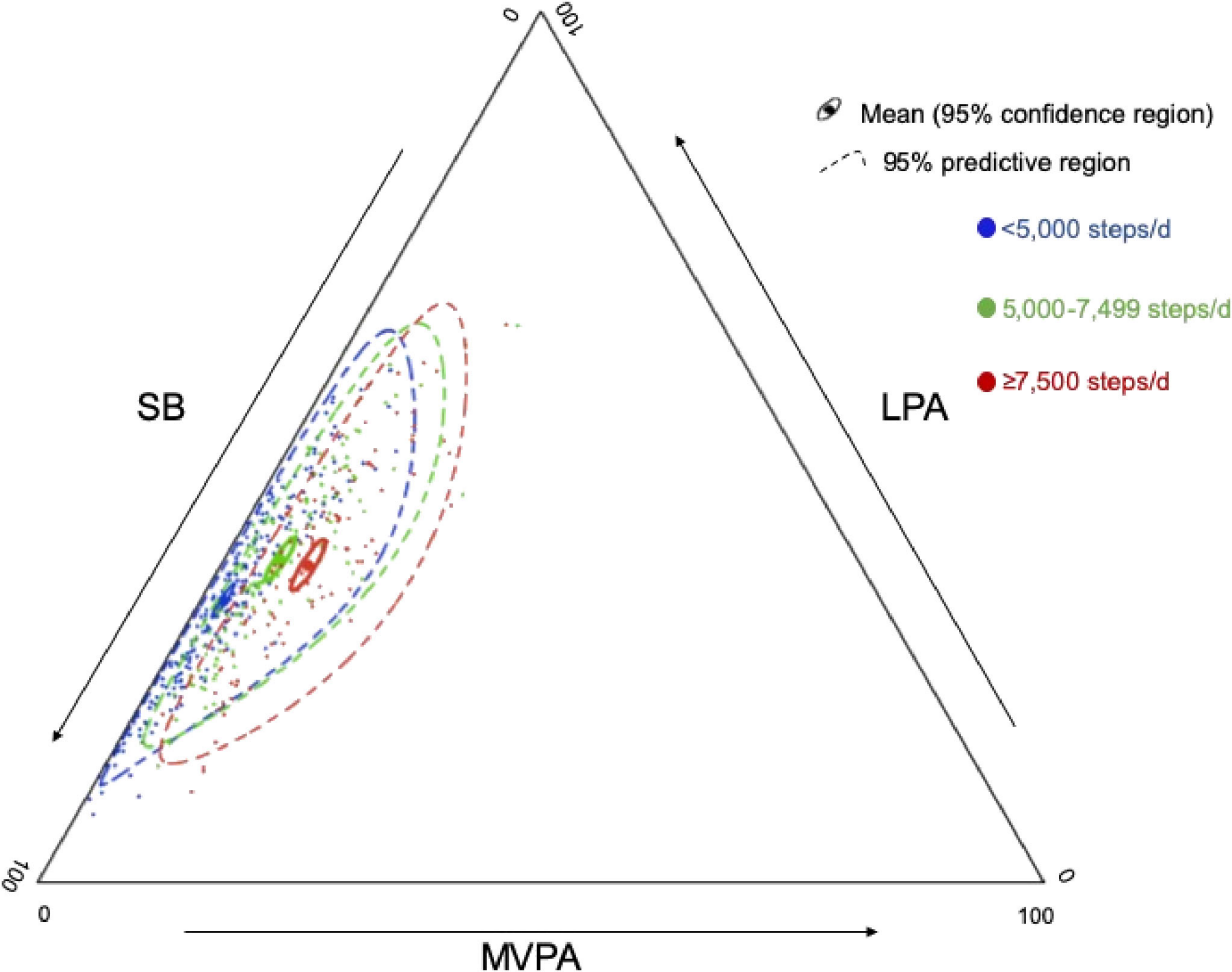
Ternary diagrams of the compositions of time spent in intensities of physical activity and sedentary behaviors by step-based categories. LPA, light-intensity physical activity; MVPA, moderate-to-vigorous physical activity; SB, sedentary behavior.

Overall, mean accelerometer wear time was 874 (SD, 90.4) min/d. Participants averaged 5,412 (SD, 2,878; range, 25–6,119) steps/d and accumulated MVPA, LPA, and SB corresponding to 4.0%, 34.8%, and 61.2% of daily waking time, respectively. There was a significant difference in the composition of time spent in different intensities of physical activity and SB between groups defined by graduated daily step counts (Pillai’s Trace, *P* < 0.001). The ternary diagram presents these compositions (Figure 1). Table 2 presents the variation matrix indicating the dispersion of each component. The largest variability was observed in ratio of MVPA to SB.

**Table 2.** Variation matrix of time spent in sedentary, light-intensity and moderate-to-vigorous physical activity

	SB	LPA	MVPA
SB	0		
LPA	0.275	0	
MVPA	0.903	0.531	0

LPA, light-intensity physical activity; MVPA, moderate-to-vigorous physical activity; SB, sedentary behavior.

A value close to zero implies that the times spent in the two behaviors involved in the ratio are highly proportional.

### Linear regression models

**Figure 2.**
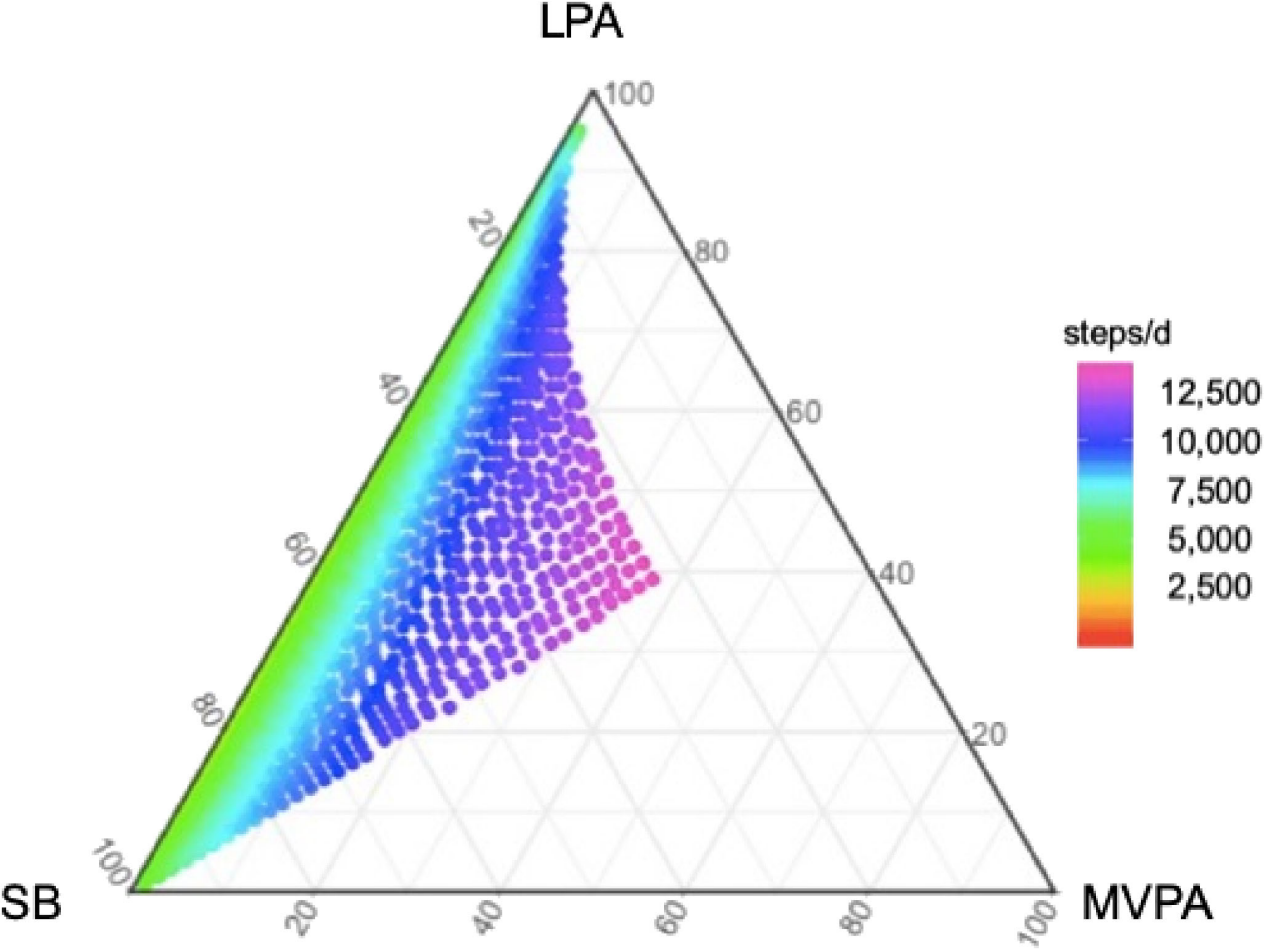
Estimated step count in proportion of sedentary behavior, light-intensity physical activity, and moderate-to-vigorous physical activity. LPA, light-intensity physical activity; MVPA, moderate-to-vigorous physical activity; SB, sedentary behavior. Note: models adjusted for age, gender, and residence of city.

Results of linear regression models with ilr coordinates are presented in Table 3. Daily step count was higher with elevated in time spent in MVPA relative to other time-stamped components (β = 2,974, *P* < 0.001) and the ratio between LPA and SB (β = 1,008, *P* < 0.001) allowing for MVPA in overall sample. The relative contribution of time spent in MVPA was significantly related to daily step count, regardless of graduated daily step count category. However, the ratio between LPA and SB had a significant relationship with daily step count in those taking <5,000 steps/d, but not in those taking 5,000–7,499 and ≥7,500 steps/d. Figure 2 shows a ternary diagram for estimated daily step count in proportion of time spent in MVPA, LPA, and SB. The relative contribution of time spent in MVPA was strongly related to daily step count.

**Table 3.** Compositional regression analysis of the relationships of sedentary and physically active behaviors with daily step count categories

	β	SE	t-value	*P*-value
	
**Overall sample**				
ilr coordinate1	2973.96	128.17	23.20	<0.001
ilr coordinate2	1007.87	270.23	3.73	<0.001
** <5,000 steps/d**				
ilr coordinate1	1210.69	91.22	13.27	<0.001
ilr coordinate2	857.95	187.81	4.57	<0.001
** 5,000–7,499 steps/d**				
ilr coordinate1	416.58	166.92	2.50	0.014
ilr coordinate2	119.54	220.76	0.54	0.589
** ≥7,500 steps/d**				
ilr coordinate1	3188.61	479.17	6.65	<0.001
ilr coordinate2	−2.67	450.98	−0.01	0.995

B, unstandardized regression coefficient estimate; ilr, isometric log-ratio; LPA, light-intensity physical activity; MVPA, moderate-to-vigorous physical activity; SB, sedentary behavior; SE, standard error.

Note: Isometric log-ratio transformation was used in compositional regression analyses. The regression estimate corresponds to one increase ilr coordinates. The ilr coordinate1 presents the ratio of MVPA time to time spent in LPA and SB, whereas ilr coordinate2 expresses the ratio of LPA time to SB time. Models adjusted for age, gender, and city of residence.

## DISCUSSION

Accounting for the co-dependence of time spent in all activity behaviors, higher daily step count was related to higher ratio of LPA to SB as well as to a greater relative proportion of time spent in MVPA, particularly among those taking <5,000 steps/d. The present study appears to be the first to examine how daily step count related to time spent in SB and LPA, after controlling for time spent in all behaviors. We also found that the relationships between daily step count and time spent in different intensities of physical activity and SB differed according to step-based categories.

Daily step count was strongly associated with MVPA, and those who accumulated more steps during the day also accumulated less time spent in LPA and SB. In general, ambulatory activities (walking or running) require moderate or higher intensity (ie, ≥3.0 METs) except in the case of very slow speeds.^21^^,^^32^ Slow walking, often observed in older people, may be captured by accelerometers as LPA. Another problem is that step count function decreases at slow walking speeds. Accelerometers used in this study have relatively accurate daily step count functions regardless of walking speed in healthy older people.^21^ However, in physically vulnerable people (therefore often less active), daily step count can be under-estimated.^21^

There have been several attempts to classify active/inactive population groups using daily step count data.^30^^,^^33^ To date, only one study investigated how daily step count related with time-stamped intensity patterns including LPA and SB.^14^ This previous study by actigraphy demonstrated that daily step count explained 25% of the variation in SB, 69% of time in LPA, and 63% of time in moderate physical activity among adults (mean age, 47 years). The differences in associations of different intensities of physical activity and SB with daily step count between these previous results and our findings may be due to methodological differences (eg, statistical methods and activity classification) and target study population. It may be difficult to directly compare findings obtained by conventional statistical analysis with those obtained by CoDa, our findings are consistent with previous study in terms daily step count is well-related with time spent in MVPA. Future research using CoDa approach is required to examine the generalizability of the present results.

There is a growing body of evidence on effects of daily step count on health outcomes.^2^^–^^4^ Recent findings from the Women’s Health Study reported dose-response relationships, with a decline in hazard ratios of mortality with more accumulated daily step count.^4^ The slope was steeper among those with accumulated lower daily step count. This is probably because they receive health benefits from increased LPA as well as MVPA along with increasing daily step count.

Daily step count is easy to measure and understand for general public. Studies over recent decades have provided important information on the national level of step-determined physical activity and its trends from around the world.^33^^,^^34^ The National Health and Nutrition Survey of Japan, for example, reported pedometer-determined daily step count in a nationally representative sample since 1995.^35^^,^^36^ Emerging evidence highlighted the potential for analyzing daily step counts automatically collected by smartphone applications for monitoring physical activity^37^^–^^39^ and for international activity comparisons^37^ with a single method. With the growing popularity of smartphones worldwide, using smartphones for physical activity and public health research may have large scale utility. Although accelerometers can provide more detailed activity patterns regarding frequency, duration, and intensity, they might be inadequate for collecting big data due to relatively high cost and low response rate from the target population (ie, selection bias). Large scale step-determined physical activity indices based on data from smartphones has considerable potential for future international comparisons. Our findings, relating daily step count to accelerometer-determined different intensities of physical activity and SB can help to inform comparisons between such studies.

### Strengths and limitations

Strengths of this study include the use of novel statistical approach (CoDa), which accounts for the compositional nature of time-use data. Our study is the novel investigation about interrelationships between daily step count and time spent in SB and different intensities of physical activity after controlling for time spent in all behaviors, which goes beyond a previous study that did not fully consider the compositional nature of time-use data.^14^ However, it has several limitations. Our sample was restricted to older adults with a narrow age range. Further research for the broader adult and older adult population are needed to evaluate the generalizability of these results. Another limitation is that the accelerometers used in this study do not detect posture accurately (eg, sitting and standing). Therefore, LPA and SB may have been over/underestimated. However, it has been reported that accelerometers used in this study slightly (25 min/d) underestimated SB time compared to inclinometer (activPAL) as a criterion.^22^ The algorithm for daily step count and time spent in different intensities of physical activity and SB can be different between activity device brands. Future research using different activity devices is required to test external validity.

In conclusion, higher daily step count can be an indicator of not only larger relative contribution of time spent in MVPA, but also higher ratio between LPA and SB, particularly among those who are the least physically active. This indicates daily step count can be useful for indirect estimate of sedentary and different intensities of physical activity patterns. Increasing time spent stepping should contribute both to reducing SB and increasing LPA. Our findings are also helpful for interpreting interrelationships between different physical activity research findings, and for better aligning pedometer-based data with accelerometer-based data.
